# New insight in massive cerebral infarction predictions after anterior circulation occlusion

**DOI:** 10.1038/s41598-023-50175-4

**Published:** 2023-12-27

**Authors:** Jingshu Chen, Mingyu Zou, Nan Zhang, Shouliang Qi, Benqiang Yang, Libo Zhang, Lin Shi, Yang Duan

**Affiliations:** 1 Department of Radiology, Center for Neuroimaging, General Hospital of Northern Theater Command, 83 Wenhua Road, Shenhe District, Shenyang, 110016 Liaoning China; 2 Department of Radiology, General Hospital of Northern Theater Command, Shenyang, China; 3 College of Medicine and Biological Information Engineering, Northeastern University, Shenyang, China; 4 Northern Theater Command Postgraduate Training Base of China Medical University General Hospital, Shenyang, China

**Keywords:** Neuroscience, Diseases, Medical research, Risk factors

## Abstract

To predict massive cerebral infarction (MCI) occurrence after anterior circulation occlusion (ACO) by cASPECTS-CTA-CS (combined ASPECTS and CTA-CS). Of 185 cerebral infarction patients with the ACO, their collateral circulation scores from CT angiography (CTA) images in two groups (MCI and non-MCI) were evaluated using Alberta Stroke Program Early CT Score (ASPECTS) and CT angiography collateral score (CTA-CS) approaches. The cASPECTS-CTA-CS was validated internally using the bootstrap sampling method with 1000 bootstrap repetitions and compared to CTA-CS. Receiver-operating characteristic curve (ROC), clinical impact curve (CIC), and decision curve analysis (DCA) strategies were used to assess the clinical practicality and predictability of both approaches (cASPECTS-CTA-CS and CTA-CS). Using net reclassification improvement (NRI) and integrated discrimination improvement (IDI) analyses, discrimination levels of the cASPECTS-CTA-CS were compared with CTA-CS. Classification and regression tree (CART) analyses was conducted to identify the best predictive values and identify subgroup of MCI. The discrimination ability of collateral circulation evaluation score using the cASPECTS-CTA-CS [AUC: 0.918, 95% confidence interval (CI): 0.869–0.967, *P* < 0.01; NRI: 0.200, 95% CI: −0.104 to 0.505, *P* = 0.197; and IDI: 0.107, 95% CI: 0.035–0.178, *P* = 0.004] was better than CTA-CS alone (AUC: 0.885, 95% CI: 0.833–0.937, *P* < 0.01). DCA indicated the net benefits of the cASPECTS-CTA-CS approach was higher than CTA-CS alone when the threshold probability range over 20%. CIC analyses showed that the number of high risks and true positives were in agreement when the threshold probability > 80%. Less than 23 of cASPECTS-CTA-CS by CART was important factor in determining MCI occurrence, and ASPECTS < 7 was followed factor. The cASPECTS-CTA-CS approach cumulatively predicted MCI after ACO.

## Introduction

Massive cerebral infarction (MCI) can be caused by unilateral occlusion of the proximal middle cerebral artery (MCA) or the internal carotid artery (ICA), with an incidence rate of approximately 10% for stroke, and 80% fatality rates^1,2^. In addition to active endovascular treatment and prevention, the timely, effective establishment, and evaluation of cerebral collateral circulation may avoid or reduce MCI events, possibly as a result of collateral circulation exerting compensatory mechanisms which increase blood supply during ischemic stroke and providing ischemic brain tissue with corresponding perfusion compensation^3–5^. With increasing importance and value of endovascular techniques in the management of acute ischemic stroke (AIS), the closer collaboration research between the vascular surgeon and the vascular radiologist need to be further improved and developed. Currently, several methods (CTA, ASPECTS, and CTA-collateral score) are used to assess and predict the occurrence of cerebral infarction^6,7^. Therefore, we think the cASPECTS-CTA-CS approach could improve MCI predictability in patients with ACO. This method will help vascular surgeon to manage acute ischemic stroke patients by evaluating cerebral collateral circulation and predicting MCI occurrence.

## Patients selection and methods

### Patient selection

**Figure 1:**
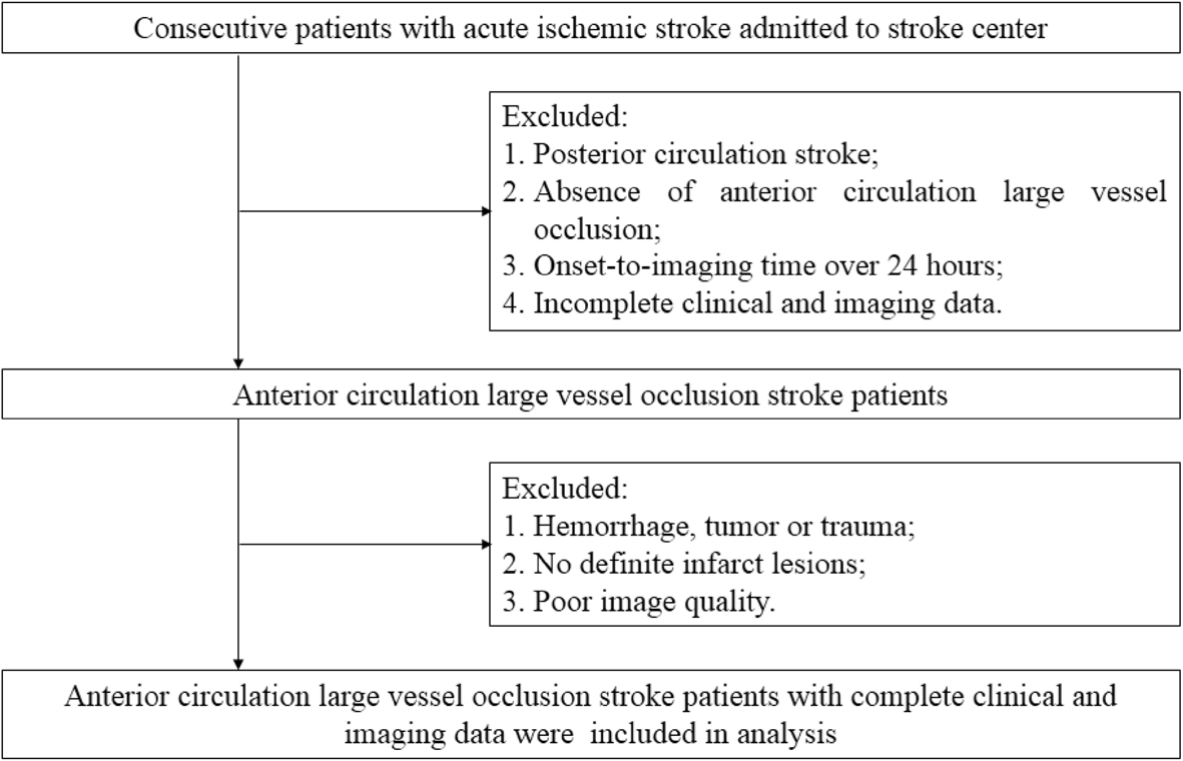
Flow diagram of screening anterior circulation large vessel occlusion stroke patients.

We retrospectively studied 185 patients with ACO. Inclusion criteria: (i) patients presented with unilateral ICA or MCA occlusion as confirmed by CTA within 24 h of onset; (ii) head CTA and routine CT exams were completed within 24 h of onset. Exclusion criteria: (i) patients with malignancy or aneurysm; (ii) baseline CT showed hemorrhagic infarction; (iii) vascular occlusion after cranial surgery; and (iv) posterior circulation infarction (Fig. 1).

Written informed consent was waived by General Hospital of Northern Theater Command Medical Ethics Committee due to the retrospective design and the absence of any intervention (reference: Y-2020-012).

### Image analysis

With in 24 h of onset, both CT and CTA were used for image analyses. ASPECTS was measured by non-contrast CT, and was used to evaluate ten anatomical sites in the MCA area to highlight ischemic alterations. An initial ASPECTS score of ten indicated no ischemic change, while one point deducted for every region affected by early ischemic changes. Thus, the ASPECTS score = 10 minus the score of all affected regions^8^. CTA-CS was measured using reconstructed maximum intensity projected CTA images: 0 = no collateral flow on the ischemic side; 1 = blood flow on the ischemic side was < 50% on the contralateral side; 2 = blood flow on the ischemic side was > 50% on the contralateral side; 3 = the ischemic side was ≥ contralateral blood flow^9^. The cASPECTS-CTA-CS scoring systems included 10 areas of ASPECTS M1–M6 area, anterior cerebral artery (ACA) blood supply area (A1–2), and posterior cerebral artery (PCA) blood supply area (P1–2). Cerebral collateral circulation perfusion in each of these areas was contralaterally compared with corresponding regions, scoring 0–3 (0 = no collateral blood flow, 1 = mild to moderate (< 50%) collateral blood flow, 2 = robust (> 50%) collateral blood flow, and 3 = normal collateral blood flow) to generate a total of 30 points^4^. MCI was defined as lesions involving > 2 lobes or an infarction area ≥ 20 cm^2^ in size^10^. Image variables were independently assessed by two experienced neuroradiologists, when disagreements arose between colleagues, a consensus was reached by a third. We graded reperfusion according to the Thrombolysis in Cerebral Infarction (TICI) scale; successful reperfusion = TICI ≥ 2b^11,12^.

According to the Ministry of Health (Ethics review on biomedical research involving human subjects), WMA (Declarations of Helsinki) and CIOMS (International ethical guidelines for biomedical research involving), all methods were performed in accordance with the relevant guidelines and regulations.

### Image acquisition

CT and CTA images from US Discovery CT 750 HD instrumentation (GE Healthcare, Milwaukee, WI, USA). CT images scanning parameters: tube current = 264 mAs, tube voltage = 120 kV, slice thickness = 5 mm, field of view = 240 × 240, and matrix = 512 × 512. CTA images scanning parameters: tube voltage = 100 kV, tube current = 120 mAs, collimator width = 40 mm, field of view = 25 cm, layer thickness and layer spacing = 5 mm. We injected 60 ml iodixanol contrast agent (270 mg/ml) at 5 ml/s via the elbow vein. Using the post-processing workstation, thin layers (0.625 mm) were constructed to generate virtual reality images depicting boneless blood vessels.

### Statistical analysis

The SPSS 22.0 package (SPSS Statistics, IBM Corporation, Armonk, New York, NY, USA) was used. Frequencies or percentages expressed categorical variables (Chi-square tests). Normally distributed continuous variables: the mean ± standard deviation (independent sample t-tests). Non-normally distributed data: the median and interquartile range (IQR) (Mann–Whitney U test). The area under the receiver operating characteristic curve (AUC) was used to check if predictability had increased for the cASPECTS-CTA-CS approach. We used decision curve analysis (DCA) and clinical impact curve (CIC) to assess if clinical practicality improved for the cASPECTS-CTA-CS approach. DCA was used to analyze all possible behaviors and outcomes by introducing threshold probabilities, thereby helping clinicians to make the best decisions for patients, and optimizing the method to generate the greatest net benefit. With the same probability, the higher the net benefit, the higher the clinical practical application value. CIC was used to establish a model to predict the risk stratification for every 1000 people, by showing the "loss: benefit", and eight scales of confidence intervals (on the X-axis).

Classification and regression tree (CART) analysis was used to divide patients into mutually exclusive subgroups, with common characteristics, by selecting optimal characteristics. CART comprised three sections: (i) feature selection; (ii) decision tree generation; and (iii) decision tree pruning, and was used to identify the best predictive value and identify specific subgroups. Variables from univariate analyses with *P* < 0 0.05 values were included in CART analyses. DCA, CIC, and CART values were plotted using R 4.1.2 (The R Foundation for Statistical Computing, Vienna, Austria). Inter-observer agreements from subjective evaluations were performed by calculating κ values (*P* < 0.05 indicated statistical significance).

### Ethics approval and consent to participate

This study was approved by the Medical Ethics Committee of the General Hospital of Northern Theater Command and agreed to be published (reference: Y-2020-012).

## Results

### Baseline characteristics

Of the 185 patients, the average age was 61.5 years and 140 were male (75.7%). Interclass correlation analyses of ASPECTS (κ = 0.855), CTA-CS (κ = 0.845), and cASPECTS-CTA-CS approach (κ = 0.839) between two observers showed excellent agreement. When compared with non-MCI patients, MCI patients displayed a higher National Institutes of Health Stroke Scale (NIHSS) (12.5 vs. 2.0), lower ASPECTS (4.0 vs. 8.0), lower CTA-CS (1.0 vs. 2.0), and significantly less alcohol drinking (46.4% vs. 51.9%) and diabetes (25.0% vs. 38.0%) levels (Table 1).

**Table 1:** Comparing clinical and imaging data between MCI and non-MCI groups.

Variables	MCI (n = 56)	Non-MCI (n = 129)	*OR*	95% *CI*	*P* value
Gender, male	41/15	99/30	1.207	0.588–2.477	0.608
Age, years	63.0 (56.0–69.0)	63.0 (55.0–67.0)	1.020	0.991–1.050	0.448
NIHSS	12.5 (8.0–16.0)	2.0 (0.5–6.0)	1.262	1.176–1.355	0.000
Initial systolic blood pressure (mmHg)	158.0 (141.3–174.8)	146.0 (134.0–163.0)	1.020	1.006–1.034	0.010
Initial diastolic blood pressure (mmHg)	89.0 (77.3–95.5)	88.0 (79.0–96.5)	0.993	0.972–1.014	0.680
Initial glucose (mmol/L)	5.9 (5.5–7.5)	5.9 (5.6–7.1)	1.044	0.929–1.173	0.841
Atrial fibrillation, n (%)	5 (8.9)	7 (5.4)	0.585	0.177–1.930	0.376
Hypertension, n (%)	30 (53.6)	71 (55.0)	1.061	0.565–1.991	0.854
Diabetes, n (%)	14 (25.0)	49 (38.0)	1.837	0.911–3.706	0.088
Smoking, n (%)	29 (51.8)	70 (54.3)	1.105	0.589–2.070	0.757
Alcohol drinking, n (%)	26 (46.4)	67 (51.9)	1.247	0.665–2.338	0.492
Coronary heart disease, n (%)	7 (12.5)	17 (13.2)	1.062	0.414–2.726	0.900
Previous cerebral infarction, n (%)	14 (25.0)	43 (33.3)	1.500	0.740–3.042	0.261
Hypercholesterolemia, n (%)	19 (33.9)	42 (32.6)	0.940	0.484–1.827	0.856
Reperfusion therapy, n (%)	14 (25.0)	27 (20.9)	0.794	0.379–1.662	0.541
Onset to CT (h)	9.5 (5.0–24.0)	14 (4.8–24.0)	0.977	0.957–0.998	0.124
Initial occlusion site, n (%)					
MCA	32 (57.1)	81 (62.8)	–	–	0.067
ICA	20 (35.7)	54 (41.9)	–	–	
MCA and ICA	14 (25.0)	10 (7.8)	–	–	
cASPECTS-CTA-CS	18.0 (16.0–21.5)	28.0 (25.5–30.0)	0.634	0.561–0.716	0.000
CTA-CS	1.0 (0.0–1.0)	2.0 (1.0–3.0)	0.084	0.039–0.179	0.000
ASPECTS	4.0 (2.0–6.0)	8.0 (7.0–10.0)	0.333	0.239–0.462	0.000

Of 41 patients who received reperfusion therapy, successful reperfusion was achieved in 28 (68.3%). MCI was identified in 14 patients (34.1%), with a median time of 3.2 h from stroke onset to the first CT (IQR = 2.700–7.250). When compared with non-MCI patients, the MCI group had lower ASPECTS (4.0 vs. 8.0), lower CTA-CS (0.0 vs. 2.0), higher NHISS (13.0 vs. 5.0), and significantly fewer occurrence of diabetes (14.3% vs. 37.0%), smoking (42.9% vs. 70.4%), alcohol drinking (50.0% vs. 63.0%), and hypercholesterolemia (14.3% vs. 33.3%) (Table 2).

**Table 2:** Clinical and imaging data of patients with reperfusion therapy.

Variables	MCI (n = 14)	Non-MCI (n = 27)	*OR*	95% CI	*P* value
Gender, male	9/5	23/4	3.194	0.696–14.664	0.130
Age, years	63.0 (52.0–65.0)	63.0 (53.0–66.0)	1.008	0.945–1.076	0.783
NIHSS	13.0 (9.8–17.3)	5.0 (2.0–8.0)	1.248	1.079–1.442	0.000
Initial systolic blood pressure (mmHg)	166.4 ± 26.6	148.3 ± 21.8	1.032	1.002–1.064	0.493
Initial diastolic blood pressure (mmHg)	93.4 ± 13.7	89.0 ± 12.1	1.029	0.976–1.085	0.729
Initial glucose (mmol/L)	6.0 (5.1–6.9)	6.1 (5.7–7.0)	0.840	0.576–1.226	0.417
Atrial fibrillation, n (%)	1 (7.1)	2 (7.4)	1.040	0.086–12.572	0.976
Hypertension, n (%)	7 (50.0)	11 (40.7)	0.688	0.188–2.520	0.576
Diabetes, n (%)	2 (14.3)	10 (37.0)	3.529	0.652–19.099	0.134
Smoking, n (%)	6 (42.9)	19 (70.4)	3.167	0.827–12.126	0.091
Alcohol drinking, n (%)	7 (50.0)	17 (63.0)	1.700	0.460–6.280	0.430
Coronary heart disease, n (%)	0 (0.0)	2 (7.4)	–	–	0.302
Previous cerebral infarction, n (%)	3 (21.4)	8 (29.6)	1.544	0.337–7.063	0.579
Hypercholesterolemia, n (%)	2 (14.3)	9 (33.3)	3.000	0.549–16.379	0.197
Initial occlusion site, n (%)					
MCA	8 (57.1)	15 (55.6)	–	–	1.000
ICA	4 (28.6)	13 (48.1)	–	–	
MCA and ICA	2 (14.3)	1 (3.7)	–	–	
Reperfusion treatment, n (%)			1.568	0.298–8.251	0.598
Venous thrombolysis	11 (78.6)	23 (85.2)			
IART	3 (21.4)	4 (14.8)			
Onset to CT (h)	3.2 (2.7–7.3)	3.7 (3.0–4.0)	1.144	0.968–1.353	0.793
Successful reperfusion (TICI ≥ 2b)	7 (50.0)	21 (77.8)	3.500	0.875–13.995	0.073
cASPECTS-CTA-CS	18.0 (15.0–20.0)	28.0 (24.0–29.0)	0.643	0.494–0.836	0.000
CTA-CS	0.0 (0.0–1.0)	2.0 (1.0–2.0)	0.097	0.023–0.404	0.000
ASPECTS	4.0 (3.0–6.0)	8.0 (7.0–9.0)	0.145	0.036–0.592	0.000

### Overall outcome

**Figure 2:**
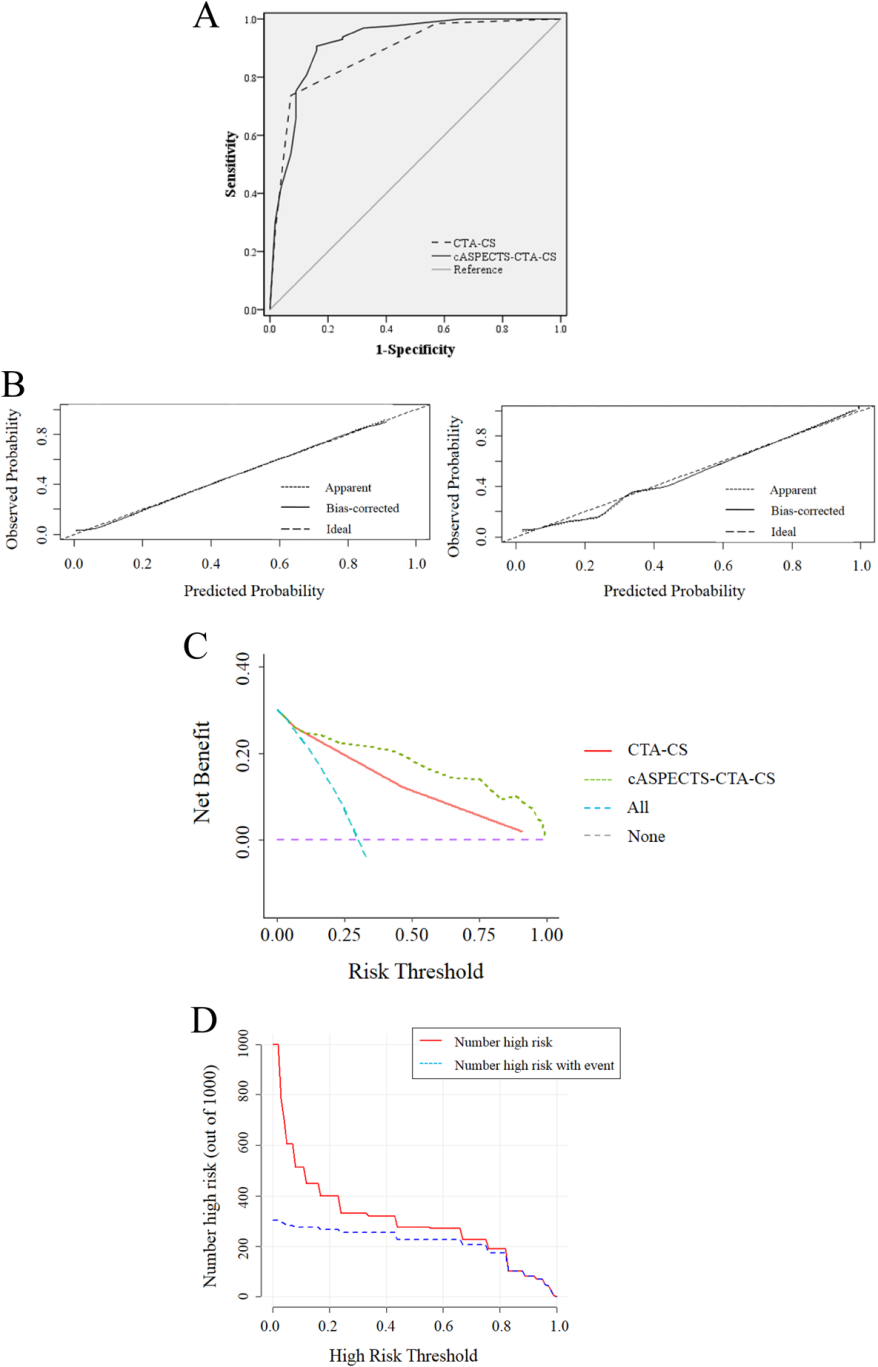
ROC, DCA, and CIC strategies were used to assess the clinical practicality and predictability of both approaches. (**A**) Receiver operating characteristic curve of CTA-CS and cASPECTS-CTA-CS to predict MCI. (**B**) Calibration plot in the CTA-CS and cASPECTS-CTA-CS. The x-axis represents the predicted probability and y-axis represents the actual probability. **(C)** Decision curve analysis comparing the clinical utility of CTA-CS alone and the c ASPECTS- CTA-CS approach. The x-axis is the threshold probability and the y-axis is the net benefit. The purple line represents that all patients are not diagnosed with MCI. The blue line represents that all patients are diagnosed with MCI. (**D**) Clinical impact curves validating the predictive value of the cASPECTS-CTA-CS approach for MCI.

The cASPECTS-CTA-CS approach AUC was 0.918 (95% CI: 0.869–0.967) and was > CTA-CS (0.885, 95% CI: 0.833–0.937) (Fig. 2A). When compared with CTA-CS (best cutoff = 1.5), the cASPECTS-CTA-CS approach (best cutoff = 22.5) had a negative predictive value of 20.3% vs. 39.5%, a specificity of 83.9% vs. 92.9%, and a sensitivity of 90.7% vs. 73.6%, which were largely different, while positive predictive value (7.1% vs. 4.0%) was slightly different. Also, the cASPECTS-CTA-CS approach improved reclassification and discrimination risks: NRI = 0.200, 95% CI: −0.104 to 0.505, and *P* = 0.197 and IDI = 0.107, 95% CI: 0.035–0.178, and *P* = 0.004. Internal validation using bootstrap resampling method with 1000 repetitions. The internal bootstrap validation calibration curve showed good agreement (Fig. 2B).

DCA demonstrated a positive net benefit of the cASPECTS-CTA-CS and CTA-CS in all threshold probabilities to predict MCI. In the threshold probability range over 20%, the cASPECTS-CTA-CS approach demonstrated higher net benefits than CTA-CS alone (Fig. 2C).

CIC analysis was used to predict the risk stratification for every 1,000 people. At each threshold probability, the number of people was classified as high risk and true positives. When the threshold probability over 80%, the number of high risks and true positives were in agreement (Fig. 2D).

### Subgroup analyses

**Figure 3:**
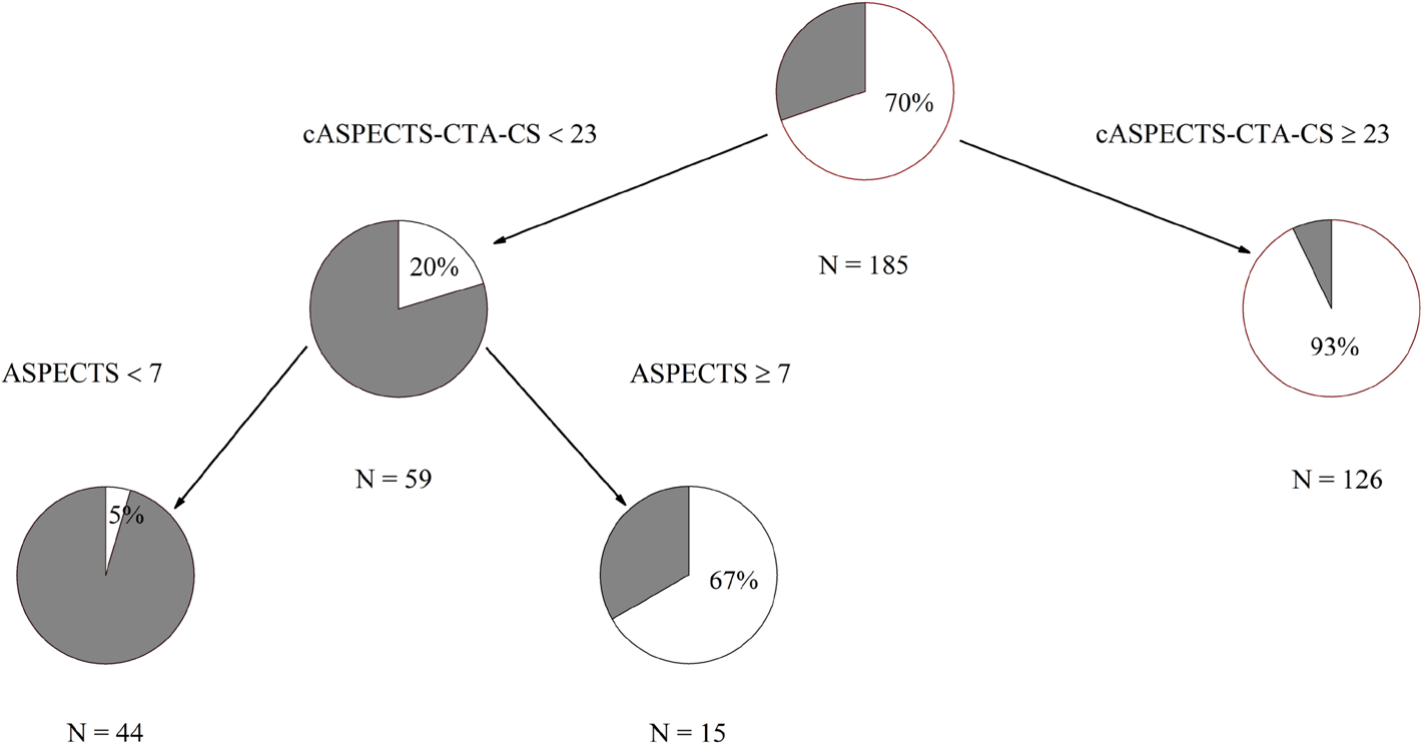
Classification and regression tree demonstrating the best variables predicting MCI. The gray area in each pie chart represents the percentage of patients with MCI in that subgroup.

Univariate analysis variables with *P* < 0.05 values underwent CART analyses (NHISS, initial systolic blood pressure, ASPECTS, CTA-CS, and the cASPECTS-CTA-CS). From 185 patients (30.3% MCI vs. 69.7% non-MCI), 59 showed cASPECTS-CTA-CS values < 23 (80.0% MCI vs.20.0% non-MCI), and 126 showed cASPECTS-CTA-CS values ≥ 23 (7.0% MCI vs. 93.0% non-MCI). Of 59 patients,44 showed ASPECTS values < 7 (95.0% MCI vs. 5.0% non-MCI) and 15 showed ASPECTS values ≥ 7(33.0% MCI vs. 67.0% non-MCI). Thus, the cASPECTS-CTA-CS, with values < 23, was important for determining MCI occurrence, while ASPECTS values were < 7 (Fig. 3).

## Discussion

We showed that score from the cASPECTS-CTA-CS approach improved the predictability of collateral circulation evaluation in MCI patients (Fig. 2). To identify specific patient subgroups with MCI by CART analysis. The cASPECTS-CTA-CS values < 23 in CART analysis was a major factor determining MCI occurrence, followed by ASPECTS values < 7. All 56 patients developed MCI. A previous study^13^ reported that patients with ASPECTS value of < 7 tended to develop MCI. This was consistent with our study, using CART analysis, we identified secondary factors in specific subgroups of patients with MCI. Collateral circulation quickly compensated for blood flow in ischemic areas, and good collateral circulation improved hypoperfusion, reduced brain tissue damage, and prolonged the survival time of the ischemic penumbra, thereby reducing infarct volume, lowering recurrence risks, and improving the prognosis.

Nine out of 126 patients with good collateral circulation (cASPECTS-CTA-CS values ≥ 23) developed MCI. Also, 47 out of 59 patients with poor collateral circulation (cASPECTS-CTA-C values < 23) occurred with MCI. However, 5 out of 15 patients with poor collateral circulation had an ASPECTS value of ≥ 7 (potentially reversible brain tissue). This phenomenon was probably due to tolerance differences in brain tissue to hypoxia in different individuals. After acute cerebral ischemia, cerebral infarction rates depend on collateral circulation compensation and the ability of tissue cells to tolerate ischemic hypoxia as brain tissue cells of different individuals have different tolerance to ischemia and hypoxia. Therefore, even if good collateral circulation is present and tolerance to ischemia and hypoxia is poor, ischemia will cause a rapid cerebral infarction, a massive infarction area, and a poor prognosis^14^.

Our analysis of patients with MCI from general clinical data showed that admission NHISS levels were related to MCI occurrence. Currently, the predictive ability of NIHSS values for MCI is widely recognized in clinical practice^15^. A related study^16^ reported that for cerebral infarction caused by internal carotid artery system occlusion, the positive predictive value of NIHSS > 9 within 3 h after onset reached 86.4%, and the positive predictive value of NIHSS of 3–7 within 6 h reached 84.4%. Other studies^17–19^ reported if the responsible lesion was in the non-dominant hemisphere and early NIHSS values were > 15, or the lesion was in the dominant hemisphere with NIHSS values were > 20 with concurrent impaired consciousness, these were classified as clinical MCI predictors. This observation was inconsistent with median admission NHISS values in 13 patients in the MCI group in our study, and may have been related to different inclusion criteria and small sample size.

Some patients with acute cerebral infarction still develop MCI, even with early reperfusion therapy. In our study, 41 patients received reperfusion therapy, of which 14 developed MCI. These results suggested that cerebral collateral circulation was the influencing factor affecting MCI occurrence after vascular recanalization in patients with acute cerebral infarction. A previous study^20^ reported that cerebral collateral circulation exerted critical roles in the pathophysiology of ischemic stroke. Poor cerebral collateral circulation accelerated progression of the infarct core, leading to cerebral edema, thus, cerebral collateral circulation may have critical roles in MCI development after revascularization. Some studies^21,22^ showed that a lower age, higher NIHSS, lower ASPECT, occlusion vessel site, cerebral collateral circulation status, and treatment method choice were associated with MCI occurrence in patients, based on hemispheric infarction with intravenous thrombolysis. We observed that MCI patients had lower ASPECTS and higher NHISS values when compared with the non-MCI group. However, lower age, occluded vessel site, and treatment method choice were not significantly associated with MCI occurrence, which may be related to different baseline data and inclusion criteria.

Our study had some limitations: (i) cerebral collateral circulation based on CTA and without DSA; (ii) only a single tertiary center with a small sample size which possibly reduced the reliability of the results. External validation of our data is required in future studies; more comprehensive factors must be examined in a large sample population to improve the predictive accuracy for MCI.

## Conclusion

In conclusion, we confirmed that new method had improved MCI predictions based on imaging results within 24 h of admission.

## Data Availability

The datasets used and analyzed during the current study are available from the corresponding author on reasonable request.

## Abbreviations

MCI: Massive cerebral infarction
NIHSS: National institutes of health stroke scale
MCA: Middle cerebral artery
ICA: Internal carotid artery
cASPECTS-CTA-CS: Combined ASPECTS and CTA-CS
CTA-CS: CT angiography collateral score
ASPECTS: Alberta stroke program early CT score
IART: Intra-arterial reperfusion therapy
TICI: Thrombolysis in cerebral infarction
